# A retrospective cohort study on the efficacy and safety of percutaneous vertebroplasty combined with bone-filling mesh container in vertebral metastases with posterior wall defect

**DOI:** 10.3389/fonc.2023.1312491

**Published:** 2024-01-12

**Authors:** Ke Zhan, Ke Chen, Guoyong Gao, Yucheng Xiang

**Affiliations:** Department of Spine Surgery, Shenzhen People’s Hospital (The Second Clinical Medical College, Jinan University; The First Affiliated Hospital, Southern University of Science and Technology), Shenzhen, Guangdong, China

**Keywords:** bone-filled mesh container, vertebroplasty, metastasis, posterior wall defect, visual analogue scale (VAS)

## Abstract

**Background:**

To evaluate the clinical safety and efficacy of percutaneous vertebroplasty (PVP) combined with bone-filling mesh containers (BFMCs) for vertebral metastases with posterior wall defect.

**Methods:**

From January 2019 to December 2021, patients with vertebral metastases and posterior wall defect who received BFMCs combined with PVP were included. The visual analog scale (VAS) scores and Oswestry disability index (ODI) scores were evaluated before and 72 hours after the operation, respectively. Post-operational X-ray and computed tomography (CT) scans were conducted to observe bone cement leakage, and complications were recorded. Follow-up CT and magnetic resonance imaging (MRI) were conducted to evaluate the condition of the operated vertebrae and the recurrence or progression of other bone metastases.

**Results:**

A total of 43 patients with 44 operated vertebrae were included. All patients successfully completed the surgery. The average VAS score decreased from 7.35 ± 0.78 to 1.63 ± 0.93 (p < 0.05), and the ODI score decreased from 80.06 ± 8.91 to 32.5 ± 4.87 (p < 0.05). Bone cement leakage was observed in 18 operated vertebrae, which were all asymptomatic. No intraspinal leakage, post-operative spinal nerve compression, pulmonary embolism, or other serious complications were recorded. A total of 21 patients had a follow-up of more than 1 year, with no operated vertebral progression, 13 target vertebrae showed obvious sclerosis and necrosis, and no adjacent pathological fracture occurred. Of these patients, 16 had different degrees of bone metastasis of other sites other than the operated vertebrae.

**Conclusion:**

For spinal metastases with posterior wall defect, PVP combined with BFMCs was highly safe and can effectively relieve pain for patients. A 1-year follow-up showed a local antitumor effect.

## 1 Introduction

The spine is one of the major sites of metastasis in patients with advanced malignancies, and its incidence increases with the course of the disease (1). The resulting skeletal-related events (SREs) seriously interfere with the subsequent antitumor treatment of patients and would even undermine survival (2). Effective surgical intervention is salvageable for these patients, and percutaneous vertebroplasty (PVP) has been clinically applied for decades with proven efficacy and safety (3). However, bone cement leakage, which may lead to patients’ paralysis or even death, remains one of PVP’s major risks. A damaged posterior wall of the vertebral body would encounter a higher risk of bone cement leakage and is thus considered a contraindication of PVP (4). With the improvement of bone cement materials and the application of new techniques in recent years, some clinicians have proposed that even in patients with vertebral body posterior wall defect, PVP could still serve as a treatment option. Bone-filling mesh container (BFMC) is one of the techniques able to control the dispersion of bone cement and reduce leakage, which has been applied in the surgical management of osteoporosis and bone metastasis. In this study, in order to evaluate the safety and efficacy of BFMCs combined with PVP, we retrospectively analyzed the application of PVP combined with BFMCs in patients with osteolytic vertebral metastasis and vertebral posterior wall defect. This study was in accordance with the STROBE reporting checklist.

## 2 Materials and methods

### 2.1 Study cohort

This was a retrospective cohort study on patients with vertebral metastases and posterior wall defect admitted to the Department of Spine Surgery of Shenzhen People’s Hospital from January 1, 2019, to December 31, 2021, who received BFMCs combined with PVP. All patients received detailed preoperative assessment to rule out exclusion criteria. Locations of vertebra metastasis were confirmed by magnetic resonance imaging (MRI) or whole body bone scan, and a further computed tomography (CT) scan was conducted to confirm the vertebra segment with posterior wall defect and the extent of damage.

The main inclusion criteria were as follows: 1) patients with thoracic vertebrae (T) 9-lumbar vertebrae (L) 5 metastasis confirmed by MRI or whole body bone scan, 2) patients’ thoracic and dorsal pain was caused by metastatic lesion and had higher than 5 points on visual analog scale (VAS) score, 3) the posterior wall destruction was confirmed by MRI or CT scan, and 4) patients with a life expectancy of over 3 months. The major exclusion criteria were as follows: 1) end-stage cancer patients and/or patients with poor cardiopulmonary, liver, and kidney function, severe anemia, or hypoproteinemia that leads to intolerance of operations; 2) patients who can only receive open surgery for decompression due to compression of the spinal cord or nerve root by pathological fracture of the vertebral body or metastatic lesion; and 3) patients with severe coagulation disorders, severe systemic infection, or skin infection at the surgical site.

### 2.2 Operation procedures

**Figure 1:**
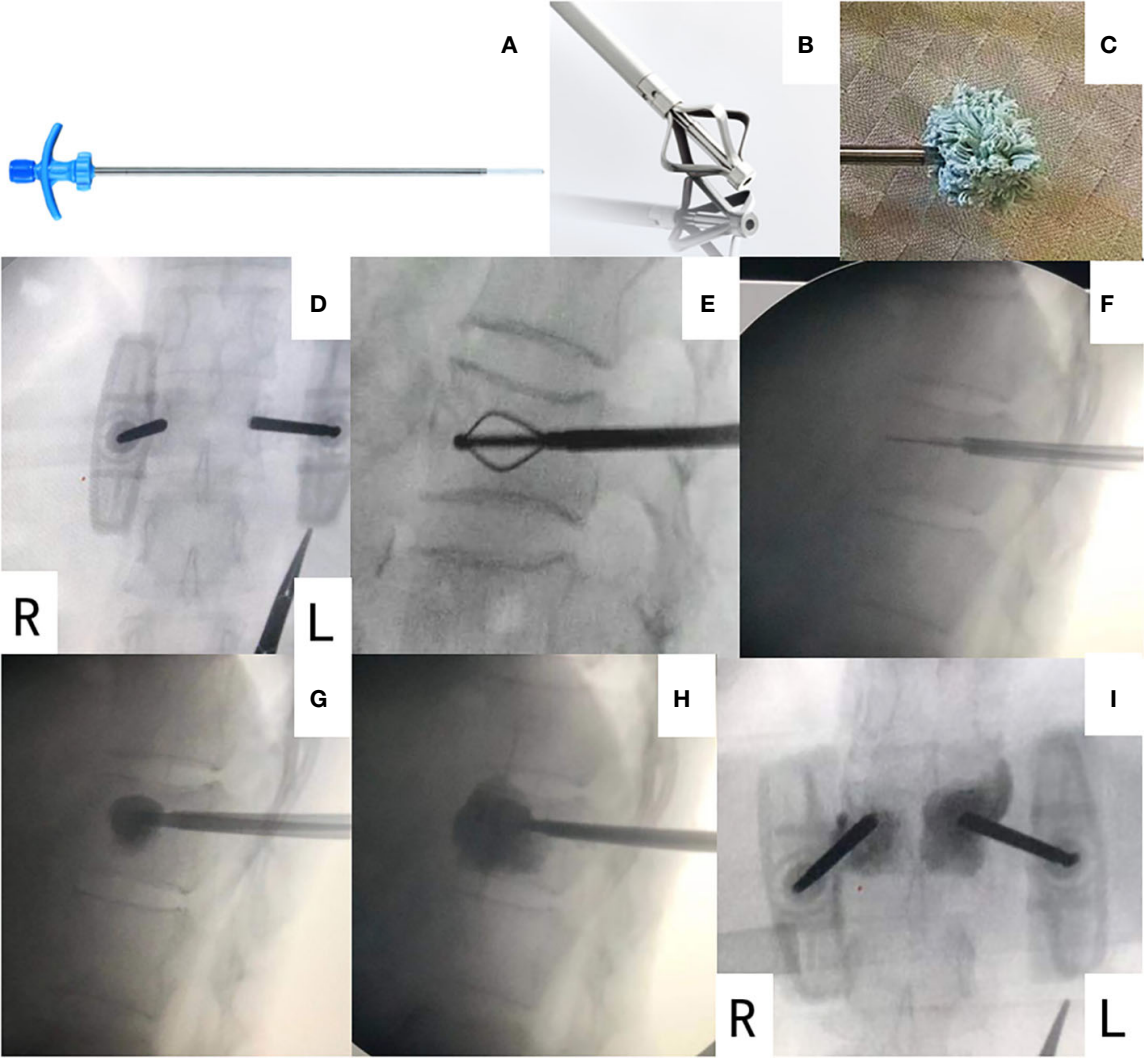
The BFMC system **(A–C)** and operation procedure **(D–I)**. **(A)** A mesh bag in compressed mode attached to a working channel. **(A)** A metal dilator. **(C)** A mesh bag filled with bone cement and in extended mode. **(D)** Locating the diseased vertebra under C-arm machine. **(E)** A metal dilator was inserted and created a space for the mesh bag. **(F)** The mesh bag in compressed mode was inserted. **(G)** 1 mL of bone cement was injected into the mesh bag, and the mesh bag was in extended open mode. **(H)** More bone cement was injected until the cement was evenly dispersed, filling two-thirds of the anterior vertebra body. PVP on the other side was conducted simultaneously. No significant leakage was observed. **(I)** Image after surgery, with BFMC on left side and PVP on right side of vertebra. BFMC, bone-filling mesh container; PVP, percutaneous vertebroplasty.

The BFMC system (Dragon Crown Medical Co., Ltd., Shandong, China) applied in this study contained one 4.0-mm puncture needle, one fine drill, one metal dilator, one screw propeller, and one bone-filling mesh bag (**Figures 1A–C**). The PVP system (Dragon Crown Medical, Shandong, China) used contained one 4.0-mm puncture needle and one pushrod. The bone cement kit (Tecres S.P.A, Sommacampagna, Italy) applied in the study mainly contained polymethyl methacrylate (PMMA), barium sulfate, and *N*,*N*-dimethyl-*p*-toluidine.

All patients were administered with local anesthesia and placed in the prone position. A biopsy of spinal metastasis was conducted before further procedures. A bilateral approach was applied to all cases. The pedicles of the vertebral arch with bone defect were located by pre-operational CT. BFMCs were applied on the side with posterior wall defect during the procedure, with simultaneous PVP on contralateral pedicles. The puncture needle was inserted through the pedicle into the targeted vertebra under the guidance of the C-arm machine fluoroscopy. Then, the core of the puncture needle was removed, and the fine drill was inserted to form a bone tunnel. Next, a metal dilator was used to create the space for the bag before the bone-filling mesh bag was inserted. At the same time, a working channel was created using the pushrod on the contralateral vertebra pedicle. Then, 1 mL of prepared bone cement was slowly injected into the mesh bag under the guidance of a C-arm machine. The mesh bag was confirmed extended open before continuing further injection of bone cement into both sides. The injection stopped until the bone cement was evenly dispersed, filling two-thirds of the anterior vertebra body without significant leakage (**Figures 1D–I**). Then, the working channel was removed, and the operation was complete.

### 2.3 Efficacy evaluation and follow-up

The VAS scores pre- and 72 hours post-operational were collected to evaluate the improvement of patients’ subjective pain symptoms. Oswestry disability index (ODI) scores were collected at the same time to evaluate the improvement of patients’ motor function status. X-ray and CT scans were performed 48 hours after the operation to evaluate the dispersion and leakage condition of bone cement. All complications after surgery, including but not limited to systemic or local infection, spinal cord compression, or bone cement implantation syndrome, were recorded in detail. Follow-up evaluation was carried out every 3 months, and the imaging study results 1 year post-operation were collected to evaluate the condition of the vertebrae. Necrosis and sclerosis of operated vertebrae and the progression of tumors on the operated and non-operated vertebrae were recorded.

### 2.4 Statistical analysis

Data were analyzed using SPSS version 20.0 software (IBM, Armonk, NY, USA). Normally distributed data are expressed as mean ± standard deviation (x ± s). Paired sample t-test was used for pre- and post-operational comparison. Count data are expressed as a percentage (%). If p < 0.05, the difference was considered statistically significant.

## 3 Results

**Figure 2:**
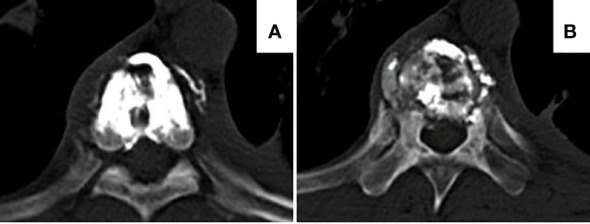
Representative images of bone cement leakage after the BFMC procedure. **(A)** Paravertebral vessel bone cement leakage. **(B)**Paravertebral bone cement leakage. Both patients had no clinical manifestation. BFMC, bone-filling mesh container.

A total of 43 patients with vertebral back wall defect from January 1, 2019, to December 31, 2021, in Shenzhen People’s Hospital were included in the study, and 44 vertebrae underwent PVP combined with BFMCs. The study cohort contained 20 patients with breast cancer, 13 with lung cancer, five with gastrointestinal cancer, and five with urinary tract malignancies (**Table 1**). The biopsy reports showed that all patients had the same malignancy originating from their primary cancer. A total of 18 thoracic vertebrae and 26 lumbar vertebrae were involved. All patients completed surgery successfully, without a record of perioperative infection or other severe adverse events. The amount of bone cement injected in both sides was 5.98 ± 1.02 mL, with a 40.9% leakage rate (18/44 vertebrae) at 48 hours after operation. However, all 18 cases had asymptomatic and non-intraspinal leakage (**Figure 2**), and no serious post-operative complications such as spinal cord nerve compression or pulmonary embolism were reported.

**Table 1:** Clinical characteristics and operated vertebrae of patients.

Clinical characteristics	Value (%)
Age (years)	58.68 ± 16.56
Gender	
Male	14 (32.6)
Female	29 (67.4)
Primary tumor (cases)	
Lung cancer	13 (30.2)
Breast cancer	20 (46.5)
Kidney cancer	5 (11.6)
Colon cancer	3 (7.0)
Gastric cancer	1 (2.3)
Hepatic cell carcinoma	1 (2.3)
Amount of bone cement injection (mL)	5.98 ± 1.02
Bone cement leakage (vertebrae)	18 (40.9)
Intraspinal	0 (0)
Intervertebral disk	4 (9.1)
Anterior/paravertebral	5 (11.4)
paravertebral vessel	9 (20.5)
Follow-up over 1 year (vertebrae)	21 (47.7)
Tumor progression of operated vertebrae	0 (0)
Sclerosis of operated vertebrae	13 (61.9%)
Tumor progression of un-operated vertebrae	16 (76.2%)
Fracture of adjacent vertebrae	0 (0)

The VAS score decreased from 7.35 ± 0.78 pre-operatively to 1.63 ± 0.93 post-operatively (p < 0.05), revealing evident post-operative pain relief (**Table 2**). The ODI score decreased from 80.06 ± 8.9 before surgery to 32.5 ± 4.87 (p < 0.05) 72 hours after surgery, showing significant improvement in patients’ motor function status.

**Table 2:** The VAS score and ODI score before and 72 hours after operation.

Score	VAS	ODI
Pre-operation	7.35 ± 0.78	80.06 ± 8.91
Post-operation	1.63 ± 0.93	32.5 ± 4.87
p-Value	<0.05	<0.05

VAS, visual analog scale; ODI, Oswestry disability index.

**Figure 3:**
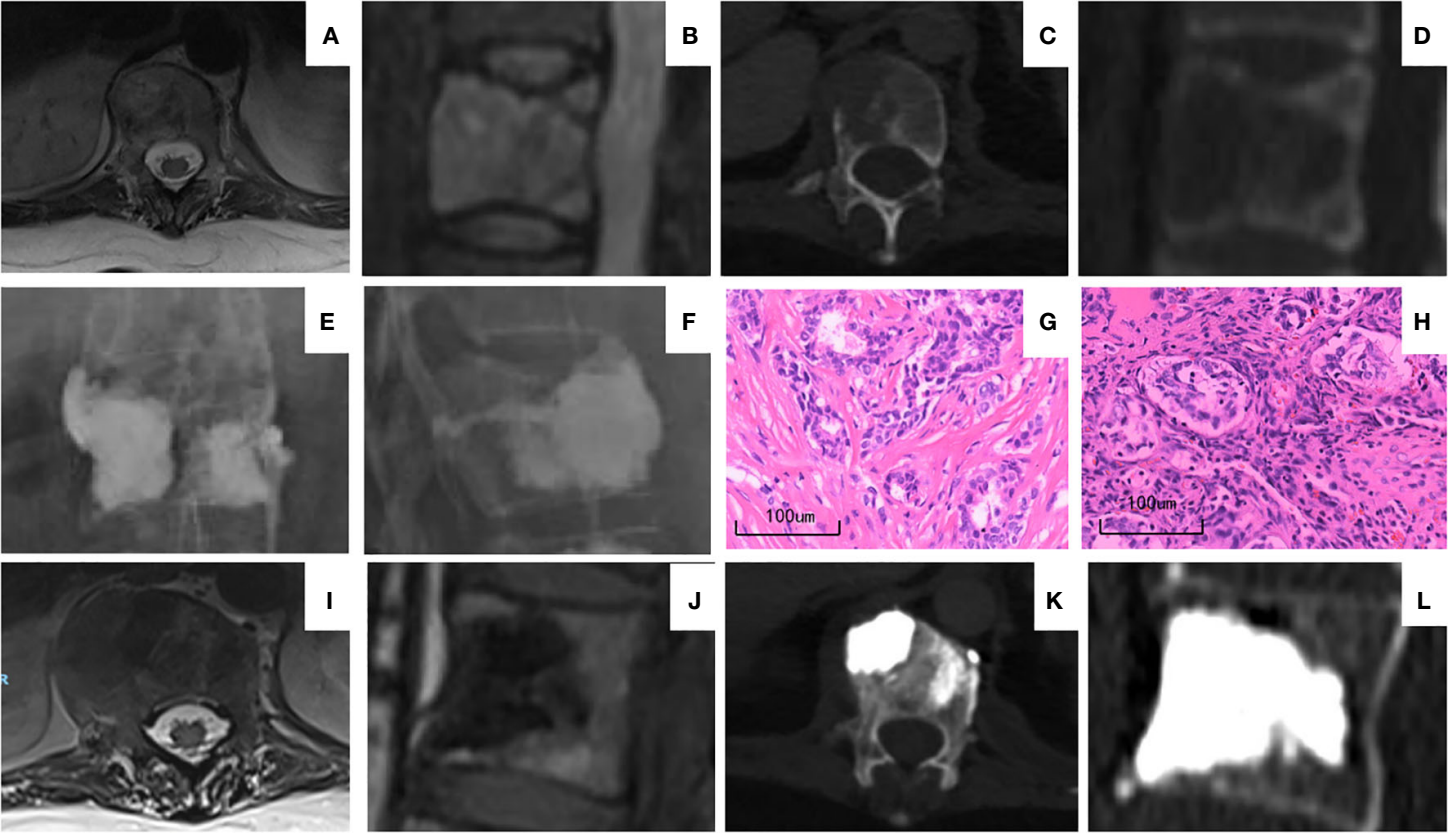
A representative case of BFMC and PVP. The patient was a 37-year-old woman with stage IV breast cancer upon diagnosis. **(A–D)** MRI **(A, B)** and CT **(C, D)** of T12 vertebra before operation showing the osteolytic destruction and pathological fracture and T12 posterior wall defect. **(E, F)** X-ray image immediately after BFMC and PVP procedure. Patient received BFMCs on the right side and PVP on left side. **(G)** H&E staining of patient’s primary tumor upon diagnosis. **(H)** H&E staining of the metastatic tumor at T12 vertebra. The pathology reports of both panels **(G, H)** were breast cancer. **(I–L)** MRI **(I, J)** and CT **(K, L)** of T12 vertebra 12 months after operation. Paravertebral vessel bone cement leakage can be seen on **(K)** with no clinical manifestation. On **(I, J)** tumor regression can be seen compared to **(A, B)** Compared to **(C, D)** sclerosis of vertebrae is shown on **(K, L)**. BFMC, bone-filling mesh container; PVP, percutaneous vertebroplasty.

The enrolled patients had a follow-up period of 7–30 months, during which 15 patients died, and 21 patients with 21 operated vertebrae had a follow-up period of over 1 year. Of these 21 patients, no progression of operated lesions or pathological fracture of adjacent vertebrae was discovered in the imaging study, and 14 cases of operated vertebrae with significant signs of sclerosis were found (**Figure 3**). A total of 17 cases reported varying degrees of bone metastasis progression on non-operated vertebrae.

## 4 Discussion

Bone metastasis is a frequently discovered complication in advanced malignancies, and the spine is the most commonly involved site, with an incidence of 30%–70% in various studies (5). Uncontrolled spinal metastasis can cause SREs including local pain, spinal cord and nerve compression, pathological fracture, and spinal instability, which would lead to paralysis and disability that severely interfere with patients’ antitumor treatment. Surgical intervention plays an important role in the integrated treatment of spinal metastasis. PVP, as a minimally invasive operation approach, has been proven valid in pain relief, spinal stability reconstruction, and local tumor control.

PVP has been applied in the treatment of hemangioma, osteoporotic fracture, and spinal metastasis since 1984, and its efficacy has long been recognized (6). It has been well accepted that compared to non-operational methods, PVP is effective in rapid pain relief, neurological function salvage, and improvement of quality of life. However, bone cement leakage, though mostly asymptomatic, sometimes could lead to vascular embolism, pulmonary embolism, intracardiac cement embolism, and intraspinal leakage, remained a non-ignorable complication (7–10). According to various literature, the leakage rate of bone cement during PVP procedures was 31.6% in osteoporotic fractures and 50%–72% in spinal metastatic cases. Vertebral posterior wall defect significantly raised the risk of bone cement leakage and has long been considered a contraindication for PVP (4, 11, 12). The traditional option for these patients was open surgery, which resulted in larger operational trauma, longer recovery, and a heavier burden in medical expenses.

With the improvement of bone cement material and operational techniques in recent years, minimally invasive operations applied in patients with vertebral posterior wall defect could achieve higher safety and efficacy (13). BFMC is one of these improved techniques. The mechanism of BFMC is to use the metal dilator to create a bone cavity before inserting a porous, multiple-layer mesh that could extend as the amount of injected bone cement increases. The bone-filling mesh bag we applied in this study was made of polyethylene terephthalate (PET) interlaced into a mesh structure, with a mesh size of 0.1–0.2 mm. Theoretically, BFMCs could control the permeate speed and direction of bone cement and thus decrease the leakage rate (14).

In an early study of applying mesh bag in symptomatic vertebral compression fractures (14), 29 patients with osteoporosis, high-impact trauma, myeloma, or tumor metastasis were enrolled, and this novel approach decreased the average pain score from 8.72 ± 1.25(SD) to 3.38 ± 2.35. Meanwhile, the average mobility score before treatment was 2.31 ± 1.94, and after treatment, it was 0.59 ± 1.05 (p < 0.001). Therefore, BFMCs offered statistically significant benefits in improvements of pain and mobility. He et al. compared the efficacy and safety of BFMCs and simple percutaneous balloon kyphoplasty in the treatment of osteoporotic vertebral compression fractures (15). The study discovered that both methods can relieve pain effectively and correct the Cobb angle; however, the bone cement leakage rate of any region, including the spinal cord, paraspinal vein, and adjacent vertebral soft tissue, was significantly lower in the BFMC group. In another retrospective cohort study on the clinical efficacy of BFMC combined with posterior screw and rod internal fixation in the treatment of thoracolumbar metastases (16), this combinational procedure had a lower bone cement leakage rate compared to the control group that received routine vertebroplasty combined with posterior spinal internal fixation (14.29% *vs.* 31.43%). Meanwhile, both groups had comparable capacity in pain control, vertebral height restoration, and mobility improvement. The application of Mesh-Hold™ bone-filling container for the treatment of pathological vertebral fractures due to osteolytic metastases was evaluated in a retrospective study (17), in which 36 patients with 105 segments were recorded. The results showed that VAS scores and ODI decreased significantly after surgery, and the bone cement leakage rate was 16.2%.

In our study, the bilateral procedure of PVP in combination with BFMCs effectively relieved the pain for patients, as the VAS score decreased from 7.35 ± 0.78 to 1.63 ± 0.93 (p < 0.05) after the operation. A total of 18 cases of bone cement leakage were discovered 48 hours post-operational CT, including four cases with intervertebral disk leakage, five anterior/paravertebral leakage, and nine vessel leakage. No symptomatic or intraspinal leakage was found in our study. There was no reported intraspinal bone cement leakage, probably due to the application of a second-generation mesh bag, which could lower the pressure of outer layer bone cement more efficiently, and the careful selection of the site of the bone cavity. During the procedure, we usually prefer to create the bone cavity at the anterior part of the vertebral body, which could avoid further destruction from the dilator to the vertebral back wall and also keep a longer distance between the mesh bag and vertebral back wall. The efficacy and leakage rates of other regions were comparable to those of previous studies.

Bilateral approaches were adopted in our study, which included BFMCs for the sides with vertebral back wall defect that were confirmed according to pre-operational CT and simultaneous PVP on contralateral pedicles. The reason for choosing the bilateral over unilateral approach was to achieve a higher rate of bone cement filling and at the same time lower the risk of leakage. It remained controversial whether a unilateral or bilateral approach was more appropriate. In the scenario of patients with osteoporotic fractures, some studies revealed that the unilateral approach had the advantage of less trauma, shorter surgical time, lower puncture-related risk, lower radiation exposure, and less medical cost. In other studies, the bilateral approach showed an advantage in higher bone cement filling rate and lower leakage rate due to less amount of cement injected into each side (18–20).

However, to the best of our knowledge, there has been no study comparing the two approaches in patients with spinal metastasis. The decision was usually made depending on clinicians’ experience. PMMA bone cement processed cytotoxic effects relatively similar to that of alcohol before polymerized. During polymerization, a temperature of over 75°C can be reached, leading to degeneration and necrosis of nerve fibers within the vertebral body, which would subsequently lower the sensitivity to pain. The thermal effect can also produce cytotoxicity to cells 3 mm around it (21, 22). Based on the well-known hypothesis of “seeds and soil”, we believed that the matrix around metastatic lesions could provide the basis for tumor progression or recurrence (23). Hence, a higher filling rate of bone cement may exert space-occupying and devascularization effects, thereby better preventing the recurrence of the metastatic tumor. Based on the above theory, a bilateral approach was selected in our study cohort.

In this study, in the 21 patients whose follow-up images of over 1 year were acquired, vertebral sclerosis was observed in 13 operated vertebrae, and 16 cases of tumor progression in non-operated vertebrae were detected. No re-fracture in adjacent vertebrae was observed. These results, to some extent, proved our hypothesis of better local tumor control with a higher rate of bone cement filling. Sun et al. evaluated the effectiveness of cement augmentation on the osteolytic lesion in patients with vertebral metastasis (24). A total of 268 vertebrae with metastatic lesions underwent PVP and were followed up, and the range of the lesions at 3, 6, 12, and 18 months after surgery were recorded. Results showed a significantly higher tumor control rate in the group of patients with vertebrae lesions that had been fully filled with bone cement than the group still left with residual tumor destruction around bone cement. Z Liu et al. carried out a study of 54 cases of spinal metastasis and discovered that the cement volume, complete filling of cement, and filling rate were factors negatively related to local bone destruction progression occurring in less than 6 months (25). Patients with lower filling rates were more likely to have early bone destruction progression compared with those with higher filling rates. Roedel et al. conducted a study in 55 breast cancer patients (137 treated vertebrae) with spinal metastasis to evaluate the effectiveness of PVP on the prevention of progression or local recurrence (26). The results revealed that the rate of local tumor progression or recurrence of vertebrae that underwent PVP was 14% (19/137), while distant new bone metastases were observed in 47 out of 55 patients (86%), showing, partially, the antitumor effect of the cement. However, no statistically significant correlation between the rate of cement filling of the lesion and progression or local recurrence post-PVP was found.

Our study also has certain limitations. This was a relatively small-sample, single-arm, retrospective study, and the operation was performed under monocenter experience. Analysis based on cohort stratification was also limited due to the small sample size. Thus, the conclusions we have drawn need to be tested and verified by further studies with larger cohorts and more centers.

## 5 Conclusions

In conclusion, in this study, we conducted a retrospective cohort study on the efficacy and safety of PVP combined with BFMCs in vertebral metastases patients with vertebral posterior wall defect. Results showed the approach we applied was highly safe, which relieved pain efficiently. The local antitumor effect was observed at 1-year follow-up. Thus, PVP combined with BFMCs was a recommendable approach for cancer patients with spinal metastasis and vertebral posterior wall defect.

## Data availability statement

The original contributions presented in the study are included in the article/Supplementary Material. Further inquiries can be directed to the corresponding authors.

## Ethics statement

The studies involving humans were approved by The Ethics Commission of Shenzhen People’s Hospital. The studies were conducted in accordance with the local legislation and institutional requirements. Written informed consent for participation was not required from the participants or the participants’ legal guardians/next of kin in accordance with the national legislation and institutional requirements. Written informed consent was obtained from the individual(s) for the publication of any potentially identifiable images or data included in this article.

## Author contributions

KZ: Conceptualization, Investigation, Methodology, Writing – original draft. KC: Data curation, Investigation, Writing – review & editing. GG: Conceptualization, Supervision, Writing – review & editing. YX: Conceptualization, Formal Analysis, Methodology, Software, Writing – review & editing.
